# Integrated Transcriptomic and Proteomic Analysis of Nutritional Quality-Related Molecular Mechanisms in “Longjia”, “Yangpao”, and “Niangqing” Walnuts (*Juglans sigillata*)

**DOI:** 10.3390/ijms252111671

**Published:** 2024-10-30

**Authors:** Hailang Wang, Yue Su, Xiang Hu, Boxiao Wu, Yun Liu, Huan Kan, Changwei Cao

**Affiliations:** Department of Food Science and Engineering, College of Biological and Food Engineering, Southwest Forestry University, Kunming 650224, China; whl9264@swfu.edu.cn (H.W.); suyue@ksu.edu.cn (Y.S.); guyun440295@yaas.org.cn (X.H.); wbx1437@swfu.edu.cn (B.W.); liuyun@swfu.edu.cn (Y.L.)

**Keywords:** walnuts, transcriptomic, proteomic, nutritional quality, molecular mechanisms

## Abstract

In this study, “Longjia (LJ)” and “Yangpao (YP)”exhibited higher contents of major nutrients compared to “Niangqing (NQ)” walnuts. The combination of transcriptome and proteome by RNA sequencing and isotope labeling for relative and absolute quantification techniques provides new insights into the molecular mechanisms underlying the nutritional quality of the three walnut species. A total of 4146 genes and 139 proteins showed differential expression levels in the three comparison groups. Combined transcriptome and proteome analyses revealed that these genes and proteins were mainly enriched in signaling pathways such as fatty acid biosynthesis, protein processing in endoplasmic reticulum, and amino acid metabolism, revealing their relationship with the nutritional quality of walnut kernels. This study identified key genes and proteins associated with nutrient metabolism and accumulation in walnut kernels, provided transcriptomic and proteomic information on the molecular mechanisms of nutrient differences in walnut kernels, and contributed to the elucidation of the mechanisms of nutrient differences and the selection and breeding of high-quality walnut seedlings.

## 1. Introduction

Walnuts, renowned as one of the four major nut species, have garnered global acclaim for their nutritional value, health benefits, and exquisite taste, attracting avid consumers in Asia, North America, and Southeastern Europe [1,2]. Commonly referred to as the “longevity fruit” and the “educational fruit” [3], walnuts have been designated as a priority crop by the Food and Agriculture Organization of the United Nations. China emerges as a prominent producer and consumer of walnuts, with Yunnan Province boasting the largest production area in the country [3,4]. The two walnut species commonly cultivated for commercial nut production worldwide are the English or Persian walnut (*Juglans regia*) and the iron walnut (*Juglans sigillata*) [5]. *Juglans sigillata*, a temperate deciduous tree native to China and an ecotype of *Juglans regia*, is primarily distributed in southwestern China. Its nuts are favored by consumers and manufacturers due to their delicious taste, nutritional value, and cultural significance [6,7]. The attributes of walnuts primarily stem from their kernels, which are rich in nutrients, particularly lipids and proteins, as well as carbohydrates, vitamins, and minerals, endowing antioxidant and anti-inflammatory properties [2,8]. Furthermore, walnuts exhibit potential efficacy in enhancing cognitive function and mitigating the risk of various diseases, including dementia, cardiovascular diseases, depression, and diabetes [9].

The nutritional quality and health benefits of walnut kernels are closely tied to factors such as cultivar or genotype. Previous studies have demonstrated that cultivar (genetic factors) exerts significant influence on multiple aspects of walnut kernels, including their vitamin content, lipid composition, peroxide values, and sterol profile [10,11]. Nevertheless, these investigations have not delved deeper into the underlying molecular-level mechanisms that are responsible for these observed differences. *Juglans regia* is a globally cultivated and well-studied species, whereas research on the nutritional differences among varieties of *Juglans sigillata*, despite its centuries-long cultivation for nut production in Yunnan Province, China [5], remains extremely limited. The combined analysis of transcriptomics and proteomics constitutes a vital methodology in contemporary biological research. Its objective lies in comprehensively integrating multi-layered biological information, thereby serving as an effective tool for elucidating differences in nutritional quality and other attributes at the molecular level, encompassing gene and protein expression, regulation, and biological processes [12]. A previous study investigated the nutritional quality-related molecular mechanisms of coix seed during breeding by this technique [13]. Furthermore, nutritional quality-related molecular mechanisms in different varieties of olive [14] and tea [15] have also been investigated through transcriptomic and proteomic techniques. The combined application of transcriptomic and proteomic approaches is widespread and diverse in its utilization. However, surprisingly, there have been few reports on walnuts (*Juglans sigillata*). Despite the factual differences in nutritional quality among different walnut kernel varieties, the underlying mechanisms responsible for these discrepancies remain largely unexplored.

Therefore, this study evaluated the nutritional quality of three walnuts (*Juglans sigillata*) from Yunnan, namely Longjia (LJ), Niangqing (NQ), and Yangpao (YP), encompassing crude protein, crude fat, total sugar, amino acids, and fatty acids. Furthermore, this study analyzed and clarified the molecular mechanisms related to their nutritional quality using transcriptomic and proteomic methods, with the aim of enhancing the quality of walnuts and facilitating the selection and breeding of high-quality varieties.

## 2. Results

### 2.1. Nutritional Quality Analysis

As shown in Table 1, the crude fat content of all three walnut varieties exceeded 65%, with no statistically significant difference (*p* > 0.05). The crude protein content was above 13%, with LJ having the highest content, followed by YP, both significantly higher than NQ (*p* < 0.05). Total sugar content was significantly higher in NQ compared to LJ and YP. Most amino acids and the primary fatty acid (linoleic acid) were present in higher amounts in LJ and YP than in NQ. These results indicate that LJ and YP possess superior primary nutritional components compared to NQ.

### 2.2. Transcriptomic Differential Analysis Among Three Walnut Varieties

#### 2.2.1. Data Quality Control and Sequence Alignment Analysis

**Figure 1:**
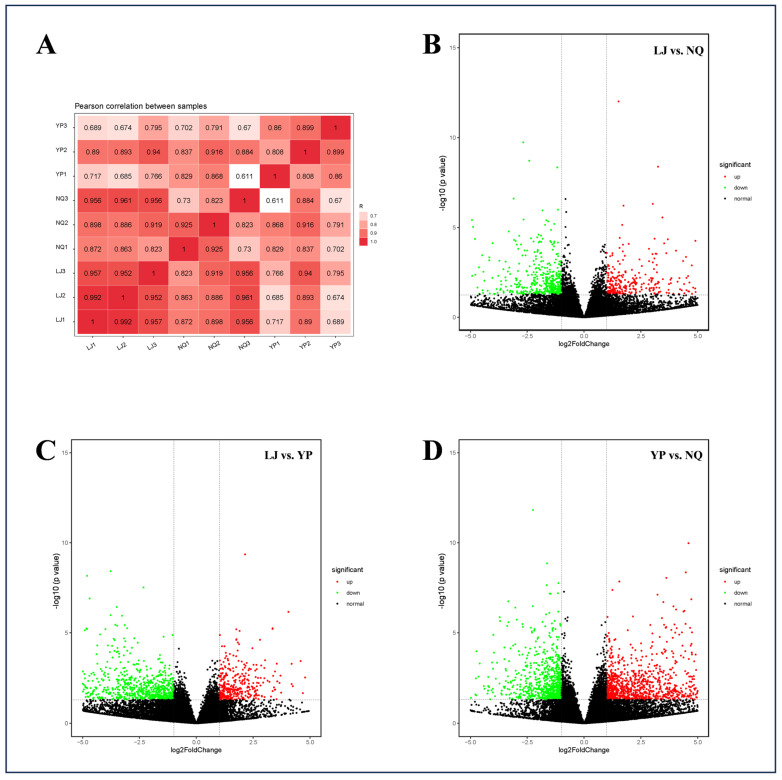
Correlation heatmap of gene expression profiles in three varieties of walnuts (**A**); volcano plots depicting the identification of DEGs among different varieties ((**B**): LJ vs. NQ; (**C**): LJ vs. YP; (**D**): YP vs. NQ).

Employing the RNA-Seq technique, transcriptome sequencing was performed on three walnut samples, resulting in raw reads. Following the removal of redundant sequences, a total of 59.36 G of clean reads were obtained, with each sample’s clean data exceeding 5.33 G. The Q20 base percentage was above 99.99%, the Q30 base percentage was above 98.22%, and the GC content ranged from 46% to 48.5%. These metrics indicate high-quality sequencing of the walnuts, enabling subsequent analysis. Comparative analysis of the filtered clean data with the reference genome revealed mapped read proportions exceeding 95.46%. Correlation analysis of the gene expression heatmap (Figure 1A) demonstrated strong correlations among the three walnut varieties (Pearson correlation coefficient R^2^ ≥ 0.611), confirming the effectiveness of the sample selection.

#### 2.2.2. Differential Expression Gene (DEG) Analysis

A volcano plot was generated using the criteria of |log2FC| > 1 and *p* < 0.05, revealing a total of 4146 differentially expressed genes (DEGs) across the three walnut varieties. Specifically, 907 DEGs were identified in the LJ vs. NQ comparison (Figure 1B), 1249 in LJ vs. YP (Figure 1C), and 1990 in YP vs. NQ (Figure 1D). Detailed information on these DEGs is provided in Supplementary Table S1. Genes related to amino acid synthesis, fatty acid metabolism, protein junctions and transport, repair of damaged proteins, cell cycle, and transcription factors differed significantly among the three comparison groups.

#### 2.2.3. GO Enrichment Analysis of DEGs

**Figure 2:**
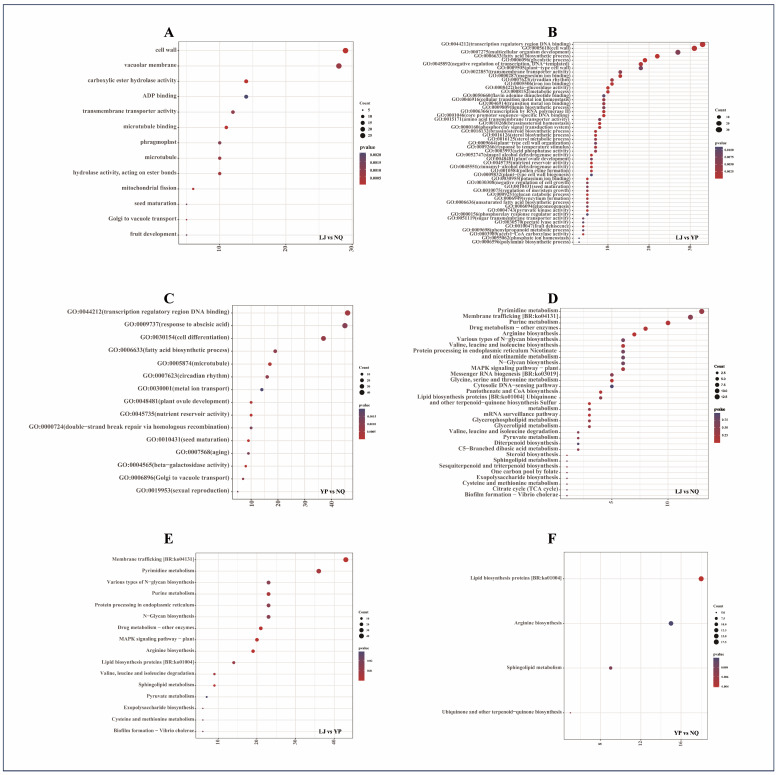
GO and KEGG enrichment results of DEGs. The GO enrichment results for the comparisons LJ vs. NQ, LJ vs. YP, and YP vs. NQ are presented in (**A**–**C**), respectively. The KEGG enrichment results for the same comparisons are shown in (**D**–**F**), respectively.

The DEGs from the three comparison groups underwent GO functional annotation analysis, with the results presented in Figure 2. In the LJ vs. NQ group, DEGs were significantly enriched in 13 GO terms, including carboxylic ester hydrolase activity, microtubule binding, Golgi to vacuole transport, seed maturation, fruit development, and ADP binding (Figure 2A). In the LJ vs. YP group, DEGs were prominently enriched in 51 GO terms, such as core promoter sequence-specific, DNA binding, glycolytic process, fatty acid biosynthetic process, glucan catabolic process, transcription by RNA polymerase II, unsaturated fatty acid biosynthetic process, amino acid transmembrane transporter activity, and nutrient reservoir activity (Figure 2B). In the YP vs. NQ group, DEGs were significantly enriched in 15 GO terms, including transcription regulatory region DNA binding, fatty acid biosynthetic process, nutrient reservoir activity, seed maturation, and metal ion transport (Figure 2C).

#### 2.2.4. KEGG Enrichment Classification of DEGs

The DEGs from the three comparison groups were annotated to KEGG pathways using the KEGG database, with the results presented in Figure 2. In the LJ vs. NQ group, DEGs were significantly enriched in 33 signaling pathways, including arginine biosynthesis, valine, leucine, and isoleucine biosynthesis/degradation, glycine, serine, and threonine metabolism, lipid biosynthesis proteins, glycerophospholipid metabolism, glycerolipid metabolism, and MAPK signaling pathway-plant (Figure 2D). In the LJ vs. YP group, DEGs were prominently enriched in 16 pathways, such as protein processing in endoplasmic reticulum, arginine biosynthesis, lipid biosynthesis proteins, valine, leucine, and isoleucine degradation, sphingolipid metabolism, N-glycan biosynthesis, various types of N-glycan biosynthesis, MAPK signaling pathway-plant, and cysteine and methionine metabolism (Figure 2E). In the YP vs. NQ group, DEGs were significantly enriched in pathways including lipid biosynthesis proteins, arginine biosynthesis, sphingolipid metabolism, and ubiquinone and other terpenoid-quinone biosynthesis (Figure 2F).

### 2.3. Proteomic Differential Analysis Among Three Walnut Varieties

#### 2.3.1. Protein Identification Information

**Figure 3:**
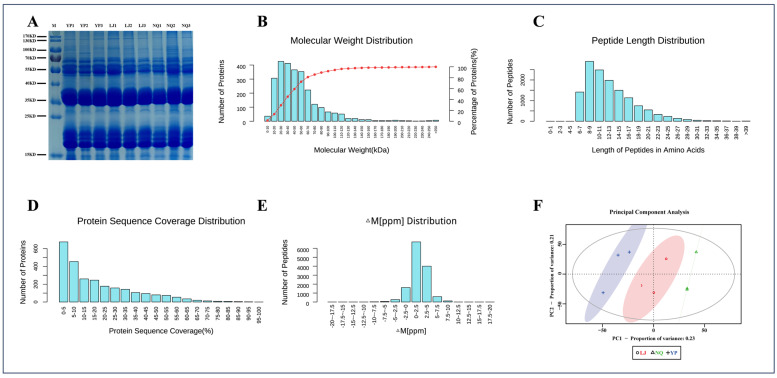
SDS-PAGE results of the samples (**A**); distribution of protein molecular weights (**B**); distribution of peptide lengths (**C**); distribution of protein sequence coverage (**D**); distribution of peptide mass deviations (**E**); principal component analysis score plot (**F**).

As illustrated in Figure 3, SDS-PAGE revealed high-quality proteins with sufficient total quantity and good parallelism (Figure 3A). Utilizing TMT quantitative technology for relative protein quantification, a total of 261,706 total spectra were acquired. Among these, 49,804 spectra were matched to identified peptides, representing 26,800 peptides, including 21,501 unique peptides. A total of 4656 protein groups were identified. The majority of proteins had molecular masses ranging from 20 to 120 kDa, with the highest abundance observed between 20 and 40 kDa (Figure 3B). The length distribution of identified peptides predominantly fell within 6 to 23 amino acids, showing a gradual decrease with increased length, indicating reasonable peptide lengths and satisfactory enzymatic digestion results (Figure 3C). The peptide coverage of identified proteins, as depicted, was relatively low, with most proteins exhibiting less than 10% coverage (Figure 3D). The mass deviation of ≥99% peptides in this project was within 8 ppm, indicating excellent instrument performance and reliable data outcomes (Figure 3E). PCA analysis revealed good intra-group clustering and distinct inter-group separation trends among the three proteomic profiles (Figure 3F).

#### 2.3.2. Differentially Expressed Protein (DEP) Analysis

**Figure 4:**
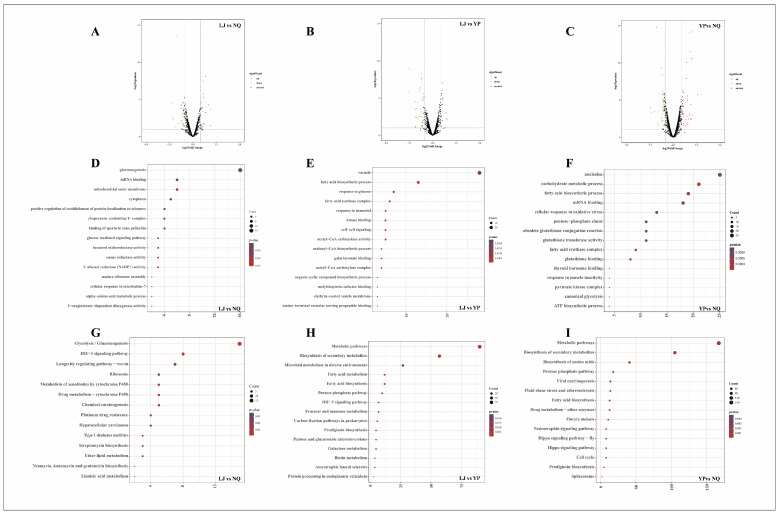
Volcano plots of differentially expressed proteins (DEPs) in the screened comparison groups: LJ vs. NQ (**A**), LJ vs. YP (**B**), and YP vs. NQ (**C**). The GO enrichment results for DEPs in these comparisons are presented in panels (**D**–**F**), respectively. The KEGG enrichment results for DEPs in the same comparisons are shown in panels (**G**–**I**), respectively.

Volcano plot analysis was performed on the identified proteins to obtain DEPs. In the LJ vs. NQ comparison, 33 DEPs were identified, with 12 upregulated and 21 downregulated (Figure 4A). For the LJ vs. YP comparison, 37 DEPs were detected, including 8 upregulated and 29 downregulated (Figure 4B). In the YP vs. NQ comparison, 69 DEPs were found, comprising 35 upregulated and 34 downregulated proteins (Figure 4C). Significant differences in proteins related to amino acid synthesis and transfer, fatty acid metabolism, riboprobe processing and transport, cell cycle, and antiviral properties were found in the three comparison groups.

#### 2.3.3. GO Enrichment Analysis of DEPs

The GO enrichment analysis of DEPs was conducted, and the results are presented in Figure 4. In the LJ vs. NQ comparison group, DEPs were significantly enriched in 102 GO terms, including gluconeogenesis, mRNA binding, glucose-mediated signaling pathway, furaneol oxidoreductase activity, enone reductase activity, 2-alkenal reductase (NADP+) activity, and alpha-amino acid metabolic process (Figure 4D). In the LJ vs. YP comparison group, DEPs were prominently enriched in 139 GO terms, such as fatty acid biosynthetic process, fatty acid synthase complex, galacturonate binding, and amino-terminal vacuolar sorting propeptide binding (Figure 4E). In the YP vs. NQ comparison group, DEPs were significantly enriched in 232 GO terms, encompassing carbohydrate metabolic process, fatty acid biosynthetic process, fatty acid synthase complex, canonical glycolysis, and glutathione transferase activity (Figure 4F).

#### 2.3.4. KEGG Enrichment Analysis of DEPs

The KEGG enrichment analysis of DEPs was performed, and the results are depicted in Figure 4. In the LJ vs. NQ comparison group, DEPs were significantly enriched in 16 signaling pathways, including glycolysis/gluconeogenesis, HIF-1 signaling pathway, ether lipid metabolism, and linoleic acid metabolism (Figure 4G). In the LJ vs. YP comparison group, DEPs were prominently enriched in 20 pathways, such as metabolic pathways, fatty acid metabolism/biosynthesis, HIF-1 signaling pathway, and protein processing in endoplasmic reticulum (Figure 4H). In the YP vs. NQ comparison group, DEPs were markedly enriched in 55 signaling pathways, including metabolic pathways, biosynthesis of amino acids, fatty acid biosynthesis, and pentose phosphate pathway (Figure 4I).

### 2.4. Integrated Transcriptomic and Proteomic Analysis

**Figure 5:**
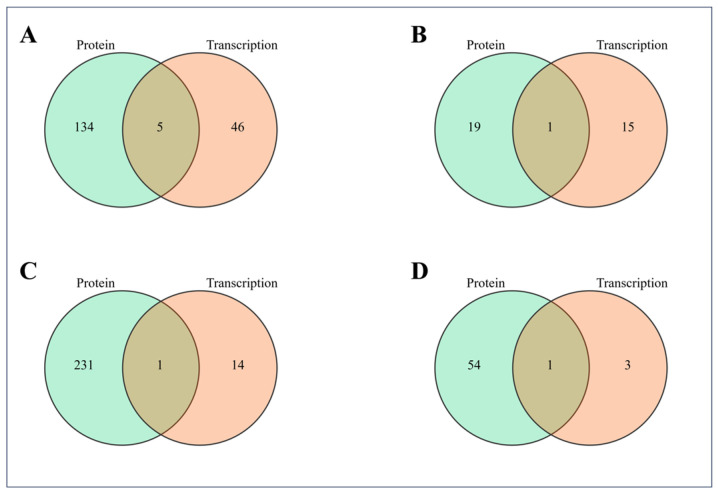
Venn diagrams displaying the commonly significantly enriched signaling pathways shared by DEGs and DEPs. DEGs and DEPs in the LJ vs. YP group significantly shared GO enrichment pathways (**A**) and KEGG enrichment pathways (**B**). DEGs and DEPs in the YP vs. NQ group significantly shared GO enrichment pathways (**C**) and KEGG enrichment pathways (**D**).

Through the combined analysis of DEGs and DEPs, it was observed that no significant common GO enrichment pathways or KEGG enrichment pathways were identified between the LJ and NQ groups. In contrast, DEGs and DEPs of the LJ vs. YP group exhibited five significantly shared GO enrichment pathways, including fatty acid biosynthetic process, iron ion binding, acetyl-CoA carboxylase activity, flavin adenine dinucleotide binding, and phenylpropanoid metabolic process (Figure 5A). Furthermore, DEGs and DEPs of the LJ vs. YP group demonstrated a significant common KEGG enrichment pathway related to protein processing in the endoplasmic reticulum (Figure 5B). For the YP vs. NQ group, DEGs and DEPs significantly shared the GO enrichment pathway of fatty acid biosynthetic process (Figure 5C). DEGs and DEPs of the YP vs. NQ group also featured a significant common KEGG enrichment pathway associated with arginine biosynthesis (Figure 5D).

**Figure 6:**
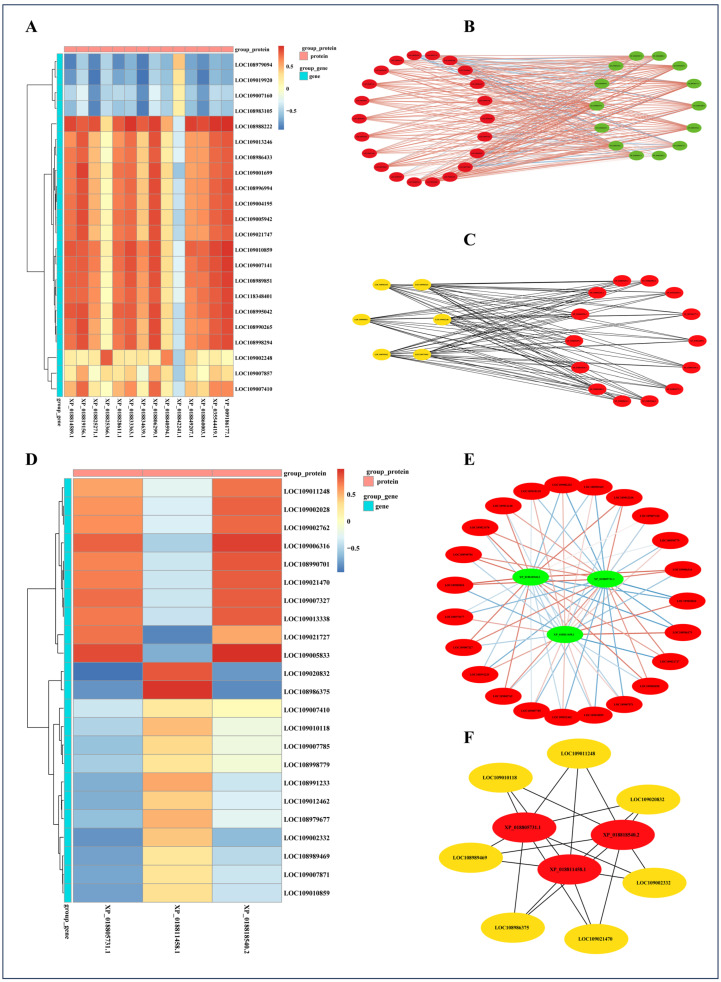
Correlation analysis of DEGs and DEPs co-significantly enriched in the “fatty acid biosynthetic process” GO signaling pathway in the LJ vs. YP comparison group (**A**); interaction network analysis (**B**) and key nodes (**C**). Correlation analysis of DEGs and DEPs co-significantly enriched in the “protein processing in endoplasmic reticulum” KEGG signaling pathway in the LJ vs. YP comparison group (**D**); interaction network analysis (**E**) and key nodes (**F**).

A correlation analysis was performed between DEGs and DEPs co-significantly enriched in the GO term “fatty acid biosynthetic process” in the LJ vs. YP group. As depicted in Figure 6A, all DEPs showed significant correlations with DEGs, except for the XP_018842241.1 protein. Figure 6B illustrates the interaction relationships between DEGs and DEPs. Through these interactions, key nodes in the walnut fatty acid biosynthetic process were identified, including XP_018814589.1, XP_018819156.1, XP_018825271.1, XP_018825366.1, XP_018828611.1, XP_018833363.1, XP_018834639.1, XP_018806299.1, XP_018840594.1, XP_018842241.1, XP_018849207.1, XP_018860003.1, XP_035544419.1, YP_009186177.1, LOC108986433, LOC108979094, LOC108983105, LOC109002248, LOC108989851, and LOC108995042 (Figure 6C).

Correlation analysis was conducted between DEGs and DEPs that were collectively and significantly enriched in the KEGG signaling pathway (protein processing in endoplasmic reticulum). High correlations were observed between XP_018811458.1 and LOC108986375, LOC109020832, and LOC109021727, as well as between XP_018818540.2 and LOC109005833, LOC108986375, and LOC109006316. XP_018805731.1 exhibited high correlations with all DEGs analyzed (Figure 6D). Through interaction analysis, key nodes in walnut fatty acid biosynthesis were identified, including XP_018805731.1, XP_018811458.1, XP_018818540.2, LOC109021470, LOC109011248, LOC109020832, LOC109010118, LOC109002332, LOC108986375, and LOC108989469 (Figure 6E,F). These findings reveal crucial proteins and genes involved in walnut fatty acid biosynthesis, protein synthesis, and processing, highlighting their essential roles in walnut growth, development, and the formation of nutritional components.

**Figure 7:**
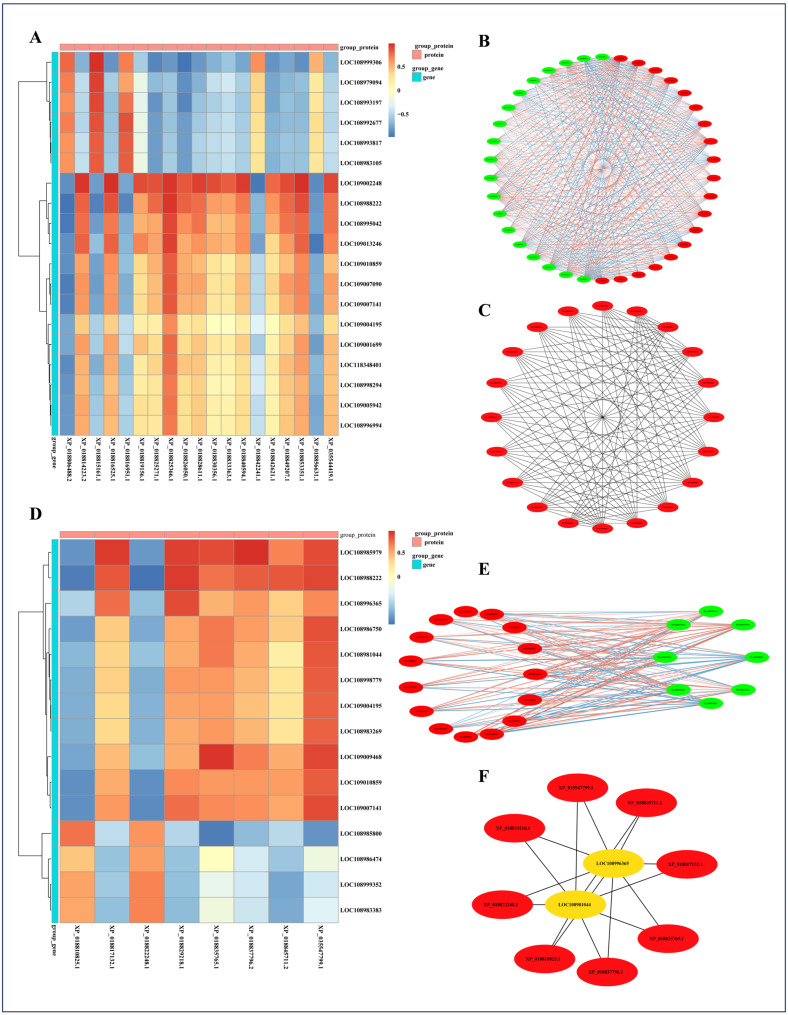
Correlation analysis of DEGs and DEPs co-significantly enriched in the “fatty acid biosynthetic process” GO signaling pathway in the YP vs. NQ comparison group (**A**); interaction network analysis (**B**) and key nodes (**C**). Correlation analysis of DEGs and DEPs co-significantly enriched in the “arginine biosynthesis” KEGG signaling pathway in the YP vs. NQ comparison group (**D**); interaction network analysis (**E**) and key nodes (**F**).

Correlation analysis was performed on DEGs and DEPs co-significantly enriched in the GO term “fatty acid biosynthetic process” from the YP vs. NQ group. High correlations were found between XP_018825366.1 and XP_018806488.2 with genes (Figure 7A). Through interaction analysis, key nodes in walnut fatty acid biosynthesis were identified, including XP_018815161.1, LOC109010859, XP_018814223.2, LOC109002248, XP_018806488.2, LOC118348401, LOC109007090, LOC108996994, XP_018830356.1, LOC109004195, XP_018828611.1, XP_018826050.1, LOC108995042, XP_018825366.1, XP_018825271.1, LOC108992677, XP_018819156.1, XP_018816951.1, LOC108998294, and XP_018816525.1 (Figure 7B,C). As in the previous section, these proteins and genes may be equally important for fatty acid synthesis in walnuts.

Correlation analysis was conducted on DEGs and DEPs that were co-significantly enriched in the KEGG pathway of “arginine biosynthesis”. High correlations were observed between LOC108988222 and both LOC108985979 and all analyzed DEPs (Figure 7D). The key nodes identified in the interaction network included XP_018810825.1, XP_018817132.1, XP_018822248.1, XP_018829218.1, XP_018835765.1, XP_018837796.2, XP_018845711.2, XP_035547799.1, LOC108981044, and LOC108996365 (Figure 7E,F). Furthermore, the interaction network diagram demonstrates a high degree of correlation among these proteins, suggesting the existence of a complex protein interaction network. These key nodes indicate their potential crucial regulatory roles in amino acid biosynthesis in walnuts, potentially interconnected through physical interactions or signaling pathways to collectively regulate walnut protein metabolism.

**Figure 8:**
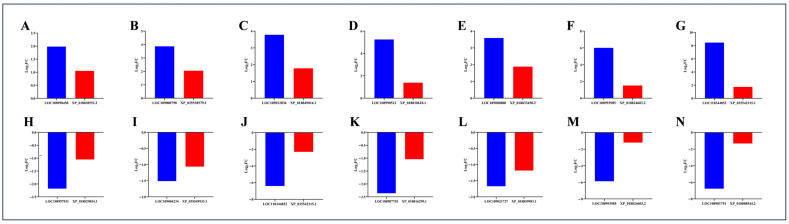
Expression trends of some genes and their corresponding proteins in the same comparison group. (**A**–**G**) indicate that genes and their corresponding proteins in the same comparison group are jointly upregulated, whereas (**H**–**N**) indicate that they are jointly downregulated. Among them, (**A**,**H**,**J**–**N**) correspond to the LJ vs. YP group, while (**B**–**G**,**I**) correspond to the YP vs. NQ group.

Moreover, as shown in Figure 8, the genes and their corresponding proteins exhibited consistent expression trends within the same comparison group, indicating that the transcriptome sequencing results and the proteome sequencing results in this study could correspond to and complement each other, with reliable sequencing results.

## 3. Discussion

In the past, walnut breeding efforts were primarily focused on achieving high yield, inadvertently leading to a decline in essential nutrients such as lipids and proteins. Nowadays, the breeding objectives for walnuts have shifted towards attaining both superior quality and high yield. However, there is a scarcity of understanding regarding the molecular mechanisms underlying these changes in nutritional composition. This study utilized transcriptomics and proteomics approaches to identify DEGs and DEPs in three walnut varieties. The signaling pathways that were significantly enriched for DEGs and DEPs together between the comparison groups included fatty acid biosynthesis, protein processing in endoplasmic reticulum, and arginine biosynthesis.

### 3.1. Fatty Acid Biosynthesis

Walnut, as a crucial woody oil crop, is characterized by its high oil content in the kernel, reaching approximately 60% [16]. The health benefits of walnut oil are attributed to its abundant polyunsaturated fatty acids (PUFAs), which are the highest among all nuts, primarily linoleic acid and α-linolenic acid [17,18]. The primary fatty acid (linoleic acid) content in LJ and YP is higher than that in NQ, while YP exhibits a higher content of the second most abundant fatty acid (oleic acid) compared to LJ (Table 1). Integrated analysis revealed that both DEGs and DEPs in the comparison groups of LJ vs. YP and YP vs. NQ were significantly enriched in the fatty acid biosynthesis signaling pathway. The shared key node DEGs and DEPs primarily included LOC109002248 (omega-3 fatty acid desaturase), LOC108995042 (acyl carrier protein 1), XP_018819156.1 (malonyl CoA-acyl carrier protein transacylase), XP_018825271.1 (enoyl-[acyl-carrier-protein] reductase [NADH]), XP_018825366.1 (SNF1-related protein kinase regulatory subunit gamma-1), and XP_018828611.1 (malonyl CoA-acyl carrier protein transacylase). LOC109002248, an omega-3 fatty acid desaturase (ω-3 FAD), is capable of introducing a double bond at the ω3 position of various ω6 fatty acids, thereby converting ω6 PUFAs into ω3 PUFAs [19]. This enzyme, ω-3 FAD, participates in the desaturation reaction of linoleic acid (C18:2) to C18:3n3, which serves as a precursor for the synthesis of long-chain polyunsaturated fatty acids (LC-PUFAs) such as eicosapentaenoic acid (EPA, C20:5n3) and docosahexaenoic acid (DHA, C22:6n3) [20]. Both EPA and DHA exhibit significant therapeutic value in clinical and epidemiological studies [21]. LOC108995042 (acyl carrier protein 1), a small acidic protein, covalently binds to fatty acyl intermediates during fatty acid elongation, serving as an essential cofactor in the process of fatty acid biosynthesis [22,23]. Malonyl CoA-acyl carrier protein transacylase (XP_018819156.1 and XP_018828611.1) serves as a pivotal enzyme in the Type II fatty acid synthesis pathway [24]. Studies have revealed that its combinatorial action with essential enzymes such as desaturases holds practical feasibility for the sustainable production of LC-PUFAs [25]. XP_018825271.1 (enoyl-[acyl-carrier-protein] reductase [NADH]) is a pivotal regulatory protein involved in fatty acid synthesis, catalyzing the final step in each elongation cycle [26].

### 3.2. Protein Processing in Endoplasmic Reticulum

Proteins, which constitute approximately 18% of walnuts, are the second most abundant component and a significant source of plant-based protein [27]. Walnut protein, a primary byproduct of walnut oil processing, is composed primarily of glutelin, glutenin, globulin, and albumin. In the past, this protein-rich byproduct has often been discarded or utilized as animal feed, resulting in a waste of valuable protein resources [28]. However, recent research has uncovered the potential benefits of walnut protein peptides in antioxidant, anti-fatigue, anticancer, and antidiabetic activities, underscoring their immense commercial value and application potential [29,30,31]. In the LJ vs. YP comparison group, both DEGs and DEPs were significantly enriched in the protein processing in endoplasmic reticulum signaling pathway. Key node DEGs and DEPs primarily comprised XP_018805731.1 (dnaJ protein homolog), XP_018818540.2 (heat shock protein 90-5), LOC109021470 (glutamine synthetase cytosolic isozyme 1), LOC109011248 (phosphoglycerate kinase 3), LOC109020832 (arogenate dehydratase/prephenate dehydratase 1), LOC109010118 (glyceraldehyde-3-phosphate dehydrogenase), LOC109002332 (asparagine synthetase), LOC108986375 (triosephosphate isomerase), and LOC108989469 (pyruvate kinase isozyme A). XP_018805731.1 (dnaJ protein homolog) and XP_018818540.2 (heat shock protein 90-5) belong to the dnaJ protein chaperone family, where chaperones facilitate the avoidance of inappropriate protein binding or aggregation in the exposed hydrophobic regions of unfolded or partially folded proteins. They guide these proteins towards productive folding and transport while also modulating non-productive interactions and aggregation with other proteins [32,33]. LOC109021470 (glutamine synthetase cytosolic isozyme 1) functions as a crucial enzyme in ammonium assimilation in higher plants, mediating the incorporation of ammonium into glutamate and yielding glutamine as the product [34]. LOC109020832 (arogenate dehydratase/prephenate dehydratase 1) represents a pivotal enzyme in the biosynthesis of phenylalanine (Phe) in plants. Phe serves as an essential constituent of proteins and a precursor for numerous plant-derived metabolites, playing a vital role in plant growth, development, reproduction, and environmental responses [35]. LOC109010118 (glyceraldehyde-3-phosphate dehydrogenase) is a pivotal enzyme involved in glycolysis, and it also interacts with proteins that participate in DNA repair [36]. LOC109002332 (asparagine synthetase), in the presence of magnesium ions, catalyzes the ATP-dependent transfer of ammonia to aspartic acid, resulting in the production of asparagine. Asparagine plays a central role in nitrogen transport and storage in plants and participates in nitrogen metabolism across various developmental stages [37].

### 3.3. Arginine Biosynthesis

The nutritional value of proteins is determined by their amino acid composition. Walnut protein is rich in essential amino acids with a well-balanced ratio [38]. In this study, walnuts contained relatively low levels of lysine and high levels of arginine, which is consistent with previous findings [39]. Research suggests that walnut consumption may act through the gut microbiota, thereby increasing endogenous production of high arginine [40]. Furthermore, walnut protein-derived arginine-containing peptides exhibit potent neuroprotective effects [41]. In the comparison between YP and NQ, both DEGs and DEPs were significantly enriched in the arginine biosynthesis pathway, with key nodes including XP_018810825.1 (glutamate dehydrogenase 1), XP_018817132.1 (aminoacylase-1-like), XP_018822248.1 (aspartate aminotransferase P2), XP_018829218.1 (aminoacylase-1), XP_018835765.1 (probable amino-acid acetyltransferase NAGS1), XP_018837796.2 (argininosuccinate lyase), XP_018845711.2 (acetylglutamate kinase), XP_035547799.1 (arginase 1), LOC108981044 (malate dehydrogenase), and LOC108996365 (aldehyde dehydrogenase family 3 member H1-like). XP_018810825.1 (glutamate dehydrogenase 1), a pivotal player in glutamate metabolism, has been recognized as a primary ammonium assimilatory enzyme and potentially involved in nitrogen remobilization during senescence [42]. XP_018829218.1 (aminoacylase-1) and XP_018817132.1 (aminoacylase-1-like) are zinc-binding enzymes involved in arginine metabolism and the methionine cycle. They catalyze the hydrolysis of acylated L-amino acids into L-amino acids and acyl groups, playing a pivotal role in the decomposition and recovery of acylated amino acids [43]. XP_035547799.1 (arginase 1) is a jasmonate-inducible protein that plays a role in polyamine production, contributing to fruit development. Furthermore, abiotic stresses can also induce the production and expression of arginase [44]. LOC108981044 (malate dehydrogenase), a pivotal enzyme in the tricarboxylic acid cycle, reversibly catalyzes the oxidation of malate to oxaloacetate by reducing NAD+ to NADH, playing a crucial role in energy balance and plant growth [45]. LOC108996365 (aldehyde dehydrogenase family 3 member H1-like) is a member of the aldehyde dehydrogenase family, which is involved in plant growth and development and plays an important role in glycolysis/glycolysis and amino acid catabolic pathways [46,47].

## 4. Materials and Methods

### 4.1. Sample Preparation

The three walnut varieties used in this study were harvested from Machang Forest Farm, Cangshanxi Town, Yangbi County, Dali, Yunnan, China (altitude: 1820 m). From each variety, three trees with robust growth, stable yield, and no pathogen infection were selected. Fruits were randomly picked from a single tree, and the kernels were aseptically extracted using sterile forceps and placed into centrifuge tubes, with approximately 1000 g of kernels from each tree individually numbered and encapsulated. These samples were immediately frozen in liquid nitrogen and stored at −80 °C for subsequent analysis.

### 4.2. Determination of Nutrient Quality

The crude protein content in walnut kernels was determined by the Kjeldahl method according to the Chinese Standard GB 5009.5-2016 [48]. The crude fat content was measured using the Soxhlet extraction method, adhering to the Chinese Standard GB 5009.6-2016 [49]. The amino acid, fatty acid, and total sugar contents were analyzed following the methodologies outlined in the Chinese Standard: GB 5009.124-2016, GB 5009.168-2016, and GB 15672-2009 [50,51,52], respectively.

### 4.3. mRNA Library Construction and Sequencing

Total RNA was extracted and purified from walnuts using TRIzol reagent (Invitrogen, Carlsbad, CA, USA) following the manufacturer’s procedure, followed by Poly(A) RNA purification via Dynabeads Oligo (dT) with double purification. Poly(A) RNA was fragmented using a Mg RNA Fragmentation Module at 94 °C. The fragments were reverse-transcribed to cDNA, and second-strand DNAs labeled with U were synthesized. A-bases were added to the blunt ends for adapter ligation. UDG enzyme was used to treat U-labeled DNAs before PCR amplification under specific conditions. The final cDNA library had an average insert size of 300 ± 50 bp. Then, 2 × 150 bp paired-end sequencing (PE150) was performed on Illumina Novaseq™ 6000 (LC Bio Technology CO., Ltd., Hangzhou, China).

### 4.4. RNA-Seq Data Processing and Analysis

Cutadapt software (cutadapt-1.9) was used to remove reads containing adaptor contamination. After removing the low-quality and undetermined bases, we utilized HISAT2 software (hisat2-2.0.4) to map the reads to the genome. The mapped reads of each sample were assembled using StringTie (stringtie-1.3.4d.Linux_x86_64) with default parameters. Subsequently, all transcriptomes from the samples were merged to reconstruct a comprehensive transcriptome using gffcompare software (gffcompare-0.9.8.Linux_x86_64). Once the final transcriptome was generated, StringTie and ballgown were employed to estimate the expression levels of all transcripts and to perform expression level analysis for mRNAs by calculating FPKM. The differentially expressed mRNAs were selected based on a |Log2(fold change)| ≥ 1 and a *p* value < 0.05. Subsequently, GO enrichment and KEGG enrichment analyses were performed on the DEGs.

### 4.5. Proteome Sequencing and Analysis

#### 4.5.1. Sample Preparation

The samples were frozen in liquid nitrogen and ground using a pestle and mortar. A 5-fold volume of TCA/acetone (1:9) was added to the powder, and the mixture was vortexed thoroughly. The mixture was then placed at −20 °C for 4 h and centrifuged at 6000× *g* for 40 min at 4 °C. The supernatant was discarded, and the precipitate was washed three times with pre-cooled acetone. The resulting precipitate was air-dried. To 20–30 mg of the dried powder, 30-fold volume of SDT buffer was added, mixed thoroughly, and boiled for 5 min. The lysate was then sonicated and boiled again for 15 min. Following centrifugation at 14,000× *g* for 40 min, the supernatant was filtered through 0.22 µm filters. The filtrate was quantified using the BCA Protein Assay Kit (P0012, Beyotime, Shanghai, China). The sample was stored at −20 °C.

#### 4.5.2. SDS-PAGE Separation

For each sample, 20 µg of proteins was mixed individually with 6× loading buffer and boiled for 5 min. Subsequently, the proteins were separated on a 12.5% SDS-PAGE gel. The protein bands were visualized through Coomassie Blue R-250 staining.

#### 4.5.3. Filter-Aided Sample Preparation

For each sample, 200 μg of proteins was incorporated into 30 μL of SDT buffer (containing 4% SDS, 100 mM DTT, and 150 mM Tris-HCl, pH 8.0). Detergent, DTT, and other low-molecular-weight components were removed using UA buffer (consisting of 8 M urea and 150 mM Tris-HCl, pH 8.5) through repeated ultrafiltration (Sartorius, Göttingen, Germany, 30 kD cutoff). Subsequently, 100 μL of iodoacetamide (100 mM IAA in UA buffer) was added to block reduced cysteine residues, and the samples were incubated for 30 min in the dark. The filters were washed with 100 μL of UA buffer three times, followed by two washes with 100 μL of 0.1 M TEAB buffer. Finally, the protein suspensions were digested overnight at 37 °C with 4 μg of trypsin (Promega, Madison, WI, USA) in 40 μL of 0.1 M TEAB buffer, and the resulting peptides were collected as filtrate.

#### 4.5.4. Tandem Mass Tag (TMT) Labeling

An amount of 100 μg peptide mixture of each sample was labeled using TMT reagent according to the manufacturer’s instructions (Thermo Fisher Scientific, Waltham, MA, USA).

#### 4.5.5. Mass Spectrometry Analysis

Each fraction was injected for nanoLC-MS/MS analysis. The peptide mixture was loaded onto the C18-reversed phase analytical column (Thermo Fisher Scientific, Acclaim PepMap RSLC 50 μm × 15 cm, nano viper, P/N 164943) in buffer A (0.1% formic acid) and separated with a linear gradient of buffer B (80% acetonitrile and 0.1% formic acid) at a flow rate of 300 nl/min. The linear gradient was 6% buffer B for 3 min, 6–28% buffer B for 42 min, 28–38% buffer B for 5 min, 38–100% buffer B for 5 min, and held at 100% buffer B for 5 min.

#### 4.5.6. Proteome Sequencing Data Processing and Analysis

Proteome Discoverer 2.2 (Thermo Fisher Scientific) software was used to convert the raw map files (.raw files) generated by Q Exactive plus into .mgf files, which were submitted to the MASCOT 2.6 server for database searching through the software’s built-in tool. Then, the lookup file (.dat file) formed on the MASCOT server was transferred back to the software through Proteome Discoverer 2.2, and the data were screened according to the FDR < 0.01 criterion to obtain highly reliable qualitative results. Mass spectrometry raw data were analyzed as raw files, and the software Mascot2.6 and Proteome Discoverer2.2 were used for library identification and quantitative analysis. According to the protein abundance level, differences of |Log2 (fold change)| ≥ 1 and a *p* value < 0.05 were deemed to be differentially expressed proteins (DEPs). All the DEPs were analyzed by GO and KEGG pathway analyses.

## 5. Conclusions

In conclusion, the main nutrient content of LJ and YP is higher than that of NQ. Combining transcriptomic and proteomic analyses as complementary approaches allowed us to investigate the mechanisms underlying the differences in the major nutrient components of three walnuts at the molecular level. A number of functional genes and proteins related to nutritional quality were identified that may contribute to nutritional quality differences among the three walnuts, especially associated with fatty acid biosynthesis, proteome processing in endoplasmic reticulum, and amino acid metabolism, which affect the synthesis, accumulation, and metabolism of nutrients in walnuts. Overall, this study provides new information on the walnut (*Juglans sigillata*) transcriptome and proteome to elucidate the molecular mechanisms underlying complex differences in walnut nutritional quality. This information will provide new references for walnut (*Juglans sigillata* and *Juglans regia*) nutritional breeding at the gene and protein levels. Nevertheless, this study lacks insight into the differences in metabolite composition among walnuts, which could potentially serve as a direction for subsequent work.

## Figures and Tables

**Table 1:** Nutritional quality differences among three varieties of walnut kernels. Different letters indicate significant differences (*p* < 0.05).

Nutritional Quality	Content		
	LJ	YP	NQ
Crude fat (g/100 g)	66.84 ± 0.68 ^a^	65.04 ± 0.31 ^a^	66.55 ± 0.48 ^a^
Crude protein (g/100 g)	16.17 ± 0.10 ^a^	15.90 ± 0.16 ^a^	13.44 ± 0.09 ^b^
Total sugar (g/100 g)	2.43 ± 0.11 ^b^	2.63 ± 0.11 ^b^	4.57 ± 0.06 ^a^
Aspartic acid (g/100 g)	0.575 ± 0.015 ^a^	0.580 ± 0.020 ^a^	0.500 ± 0.000 ^b^
Glutamic acid (g/100 g)	2.455 ± 0.025 ^a^	2.410 ± 0.060 ^a^	2.120 ± 0.070 ^b^
Serine (g/100 g)	1.205 ± 0.015 ^a^	1.185 ± 0.025 ^a^	1.025 ± 0.005 ^b^
Histidine (g/100 g)	1.390 ± 0.020 ^a^	1.350 ± 0.020 ^a^	1.160 ± 0.020 ^b^
Glycine (g/100 g)	3.040 ± 0.060 ^a^	2.955 ± 0.055 ^a^	2.425 ± 0.015 ^b^
Threonine (g/100 g)	0.400 ± 0.00 ^a^	0.395 ± 0.005 ^a^	0.350 ± 0.000 ^b^
Arginine (g/100 g)	5.205 ± 0.085 ^a^	5.060 ± 0.070 ^a^	4.400 ± 0.060 ^b^
Alanine (g/100 g)	0.770 ± 0.010 ^a^	0.760 ± 0.020 ^a^	0.700 ± 0.000 ^b^
Tyrosine (g/100 g)	0.705 ± 0.015 ^a^	0.695 ± 0.015 ^a^	0.620 ± 0.030 ^a^
Valine (g/100 g)	0.585 ± 0.005 ^a^	0.585 ± 0.015 ^a^	0.510 ± 0.000 ^b^
Isoleucine (g/100 g)	0.490 ± 0.000 ^a^	0.485 ± 0.005 ^a^	0.440 ± 0.010 ^b^
Phenylalanine (g/100 g)	0.885 ± 0.015 ^a^	0.870 ± 0.020 ^a^	0.780 ± 0.010 ^b^
Lysine (g/100 g)	0.545 ± 0.025 ^a^	0.515 ± 0.015 ^a^	0.505 ± 0.0150 ^a^
Leucine (g/100 g)	1.180 ± 0.010 ^a^	1.180 ± 0.030 ^a^	1.050 ± 0.010 ^b^
Proline (g/100 g)	0.965 ± 0.015 ^b^	1.085 ± 0.035 ^a^	0.935 ± 0.005 ^b^
Methionine (g/100 g)	0.290 ± 0.000 ^a^	0.285 ± 0.005 ^ab^	0.260 ± 0.010 ^b^
C14:0 Myristic acid (%)	0.013 ± 0.000 ^a^	0.014 ± 0.001 ^a^	0.012 ± 0.000 ^a^
C16:0 Palmitic acid (%)	6.945 ± 0.015 ^b^	7.340 ± 0.000 ^a^	4.820 ± 0.005 ^c^
C16:1 Palmitoleic acid (%)	0.208 ± 0.001 ^a^	0.201 ± 0.000 ^b^	0.081 ± 0.001 ^c^
C17:0 Margaric acid (%)	0.046 ± 0.000 ^a^	0.046 ± 0.001 ^a^	0.042 ± 0.001 ^b^
C18:0 Stearic acid (%)	2.005 ± 0.005 ^b^	1.985 ± 0.005 ^b^	2.090 ± 0.005 ^a^
C18:1 Oleic acid (%)	19.100 ± 0.000 ^c^	20.700 ± 0.000 ^b^	35.100 ± 0.050 ^a^
C18:2 Linoleic acid (%)	61.750 ± 0.050 ^a^	61.600 ± 0.000 ^a^	47.800 ± 0.050 ^b^
C18:3 Linolenic acid (%)	9.715 ± 0.025 ^b^	7.850 ± 0.000 ^c^	9.860 ± 0.000 ^a^
C20:0 Arachidic acid (%)	0.067 ± 0.001 ^b^	0.065 ± 0.001 ^b^	0.072 ± 0.001 ^a^
C20:1 Eicosenoic acid (%)	0.109 ± 0.000 ^b^	0.108 ± 0.004 ^b^	0.157 ± 0.001 ^a^
C20:2 Eicosadienoic acid (%)	0.016 ± 0.001 ^a^	0.014 ± 0.000 ^ab^	0.012 ± 0.001 ^b^
C22:0 Behenic acid (%)	0.022 ± 0.000 ^b^	0.021 ± 0.000 ^b^	0.028 ± 0.001 ^a^

## Data Availability

The data supporting the results of this study are included in the present article.

## Supplementary Materials

The supporting information can be downloaded at https://www.mdpi.com/article/10.3390/ijms252111671/s1.
